# Supplementary material for: Smartphone Video-Based Knee Extension Moments During Chair Rise Relate to MRI Measures of Muscle Force-Generating Capacity
Source: IEEE Trans Neural Syst Rehabil Eng. Author manuscript; Available in PMC 2026 Oct 19. (PMC13679900; doi:10.1109/TNSRE.2026.3728290)
Supplement: Supplementary file 1 [file NIHMS2209218-supplement-supp1-3728290.pdf]

Supplementary Material

We conducted exploratory analyses to determine how the chosen isokinetic motion and MRI-measures effected our results. In particular, we compared peak knee extension moments during eccentric, isometric, and concentric contractions to MRI-derived total volume, radial diffusivity, and composite score (Supplementary Fig. 1). The correlation strengths were higher with the faster movement, regardless of being eccentric or concentric. Also, we associated lean muscle volume (defined as total volume multiplied by water fraction) to OpenCap-derived knee extension moment and the peak knee moment measured by isometric and isokinetic dynamometry (Supplementary Fig. 2). The strengths of these correlations were similar to total muscle volume. These additional analyses were conducted to support the robustness, physiological plausibility, and methodological choices underlying the main results.

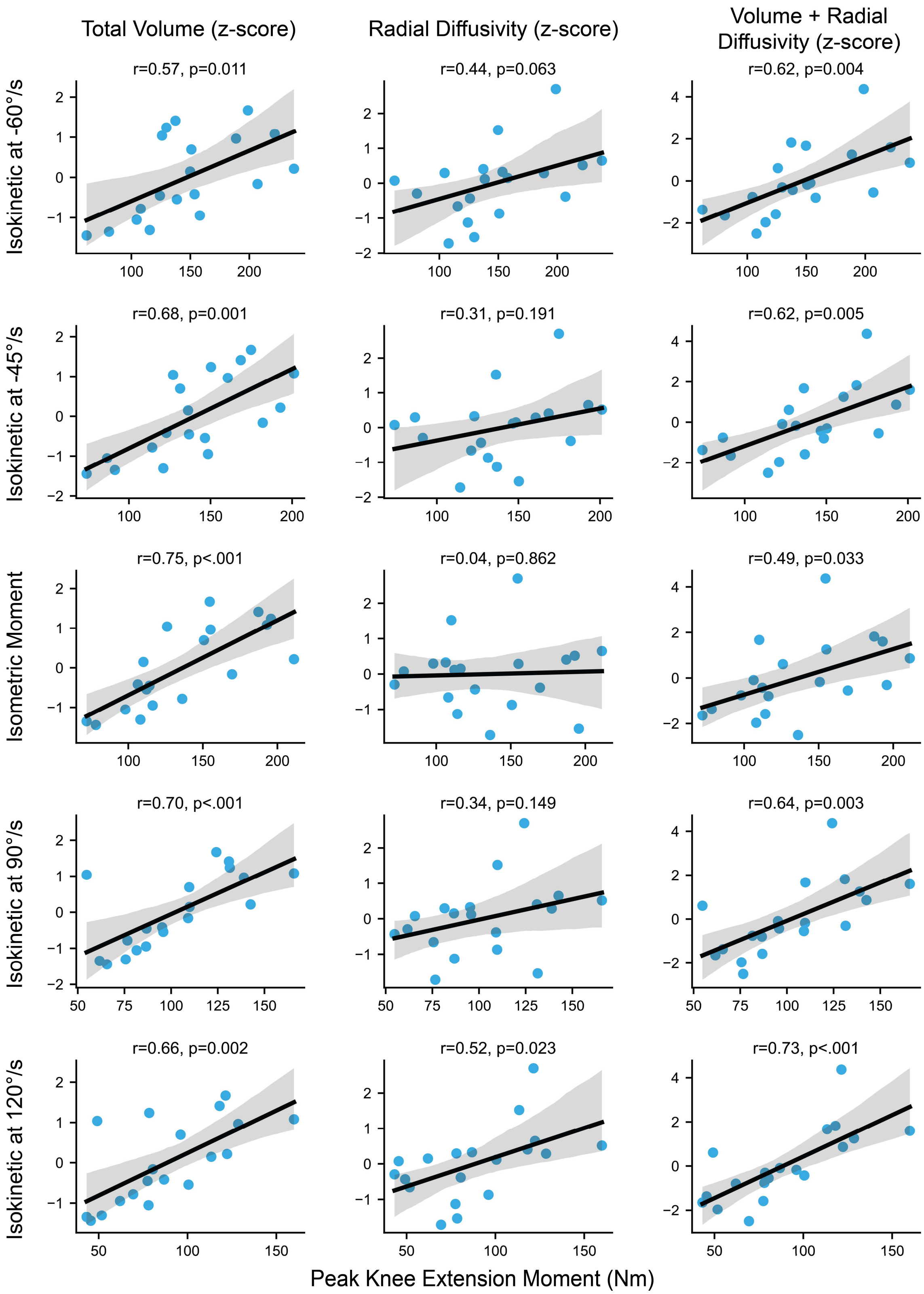

Total Volume (z-score)
Radial Diffusivity (z-score)
Volume + Radial Diffusivity (z-score)
Isokinetic at -60°/s
Isokinetic at -45°/s
Isometric Moment
Isokinetic at 90°/s
Isokinetic at 120°/s
r=0.57, p=0.011
r=0.44, p=0.063
r=0.62, p=0.004
r=0.68, p=0.001
r=0.31, p=0.191
r=0.62, p=0.005
r=0.75, p<.001
r=0.04, p=0.862
r=0.49, p=0.033
r=0.70, p<.001
r=0.34, p=0.149
r=0.64, p=0.003
r=0.66, p=0.002
r=0.52, p=0.023
r=0.73, p<.001
Peak Knee Extension Moment (Nm)

Supplementary Fig. 1: Peak knee extension moments from eccentric, isometric, and concentric contractions measured with dynamometry, correlated with MRI measures of volume and the composite MRI score. Higher velocities, both eccentrically and concentrically, correlated more strongly to radial diffusivity. Isokinetic moments at 120°/s were chosen since these contractions are maximally dynamic and are concentric, similar to the chair rise motion analyzed with OpenCap. Since this is an exploratory analysis, p-values are not corrected for multiple comparisons.

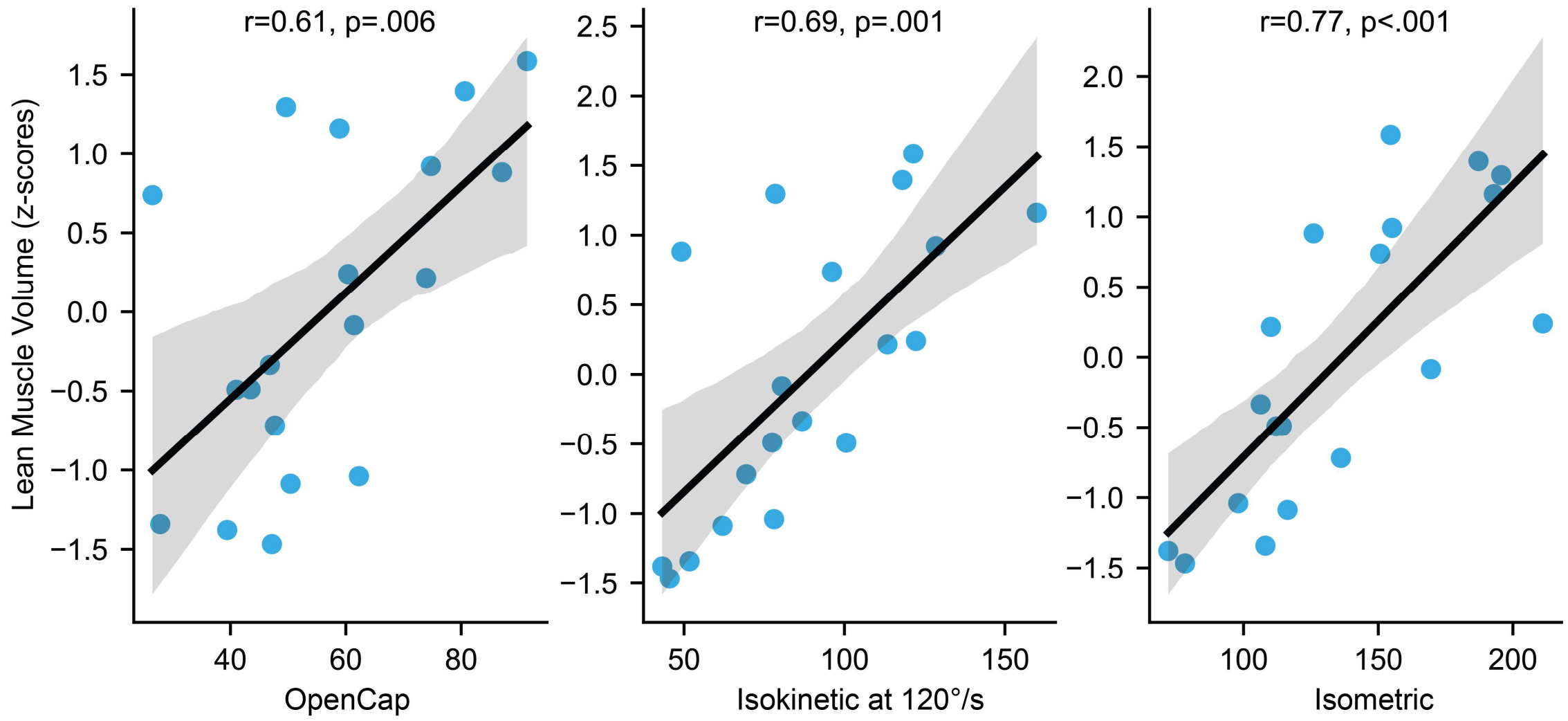

Supplemental Fig. 2: The peak knee extension moment measured with OpenCap, isokinetic dynamometry, and isometric dynamometry, correlated with MRI-measured lean muscle volume (r=0.61–0.77, p<0.01; not corrected for multiple comparisons). These associations are similar to those reported for total volume (r=0.63–0.75, p=0.002–0.014; corrected for 18 tests).
